# Subglottic Stenosis After Double‐Lumen Tube Intubation With Reintubation: A Case Report and Review of Japanese Cases

**DOI:** 10.1002/ccr3.70085

**Published:** 2025-01-06

**Authors:** Masami Suzuki, Naohiro Yoshida

**Affiliations:** ^1^ Department of Otolaryngology‐Head and Neck Surgery Jichi Medical University Saitama Medical Center Saitama Japan

**Keywords:** double‐lumen tube, reintubation, subglottic stenosis, tracheostomy

## Abstract

Subglottic stenosis after double‐lumen tube (DLT) intubation is more likely to occur when an oversized DLT, specifically a 35 Fr DLT, is used in older, shorter women. Reintubation in such cases is challenging and may cause additional traumatic laryngitis. Tracheostomy is the best management for subglottic stenosis after DLT intubation.

## Graphical abstract

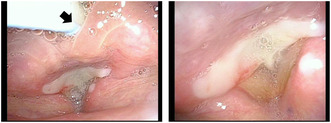

## 1 Introduction

A double‐lumen tube (DLT) is widely considered the preferred method for thoracic surgery requiring one‐lung ventilation. Compared with single‐lumen tubes, DLTs are made of harder material and have a larger outer diameter, so they have a higher incidence of postoperative sore throat and hoarseness due to airway damage [1]. In Japan, many cases of subglottic stenosis have been reported as a complication of DLT intubation at surgery. We searched for articles on subglottic stenosis after DLT intubation at surgery in English through PubMed and Google Scholar, and in Japanese through Ichushi‐Web, an online search service for Japanese medical literature. One article was published in English and 10 articles were published in Japanese [2, 3, 4, 5, 6, 7, 8, 9, 10, 11, 12]. All reported cases were from Japan (Table 1).

**TABLE 1:** List of the enrolled publications of this report and this case.

Case author	Year reference	Sex	Age	Height (cm)	Weight (kg)	Primary disease	Anesthesia time (min)	DLT size (Fr)	Resistance during intubation	Respiratory symptoms onset (POD)	Steroid administration before AM	Airway secured date (POD)	AM method	Steroid administration after AM	Improving date of SS (POD)
1. Shinjo Y	2010 [2]	F	76	155.5	52.1	LC	NA	35	+	1	mPSL	1	T	−	9
2. Ito Y	2013 [3]	F	81	148	46	LC	95	35	+	3	DEX 13.2 mg for 4 days	8	T → GR by MLS	−	NA
3. Shimagaki T	2016 [4]	F	73	146	42	LC	NA	35 → 32 → 28	+	2	mPSL in one dose	2	RI (×) → C	−	NA
4. Hayashi M	2018 [5]	F	83	149.3	57.5	LC	NA	35 → 32	+	1	mPSL 40 mg for 3 days	3	RI (○)	+	16
5. Takiwaki M	2018 [6]	F	81	142	47	LC	134	35	+	3	DEX	3	T	+	10
6. Takahashi R	2019 [7]	F	81	146.1	51.3	LC	341	35 → 32	+	2	HCSS 200 mg in one dose	2	T	−	9
7. Yonei A	2020 [8]	F	83	144.1	48.7	LC	280	35	+	3	mPSL 500 mg for 3 days	6	T	+	15
8. Sei H	2021 [9]	F	80	140	41	LC	265	32 → 28	+	NA	NA	2	T	NA	12
9. Fukui M	2021 [10]	F	80	143.6	45.4	LC	120	35	+	2	−	2	Needle C	+	8
10. Nakasumi M	2022 [11]	F	70	152.5	46.3	LC	122	35	+	2	DEX19.8 mg for 1 day	2	T	+	9
11. Oiwa H	2024 [12]	F	75	161	51	AMT	166	35	+	1	−	4	T	+	12
12. This case		F	81	142.5	42.2	LC	216	35	+	1	−	1	RI (○) → T	−	22

Abbreviations: AM, airway management; AMT, anterior mediastinal tumor; C, cricothyroidotomy; DEX, dexamethasone; DTL, double‐lumen tube; F, female; Fr, French; GR, granulation resection; HCSS, hydrocortisone sodium succinate; LC, lung cancer; MLS, microscopic laryngeal surgery; mPSL, methylprednisolone; NA, not applicable; POD, postoperative day; RI, reintubation; SS, subglottic stenosis; T, tracheostomy.

Herein, we present a case of subglottic stenosis after DLT intubation with reintubation, summarize previous reports of subglottic stenosis after DLT intubation, and discuss treatment options.

## 2 Case History

An 81‐year‐old woman was found to have an abnormal chest shadow during an annual medical examination. A chest computed tomography scan revealed a mass lesion in the lower lobe of her right lung. The patient was referred to the Department of General Thoracic Surgery of our hospital due to a strong suspicion of lung cancer. The patient's height was 142.5 cm, and weight was 42.2 kg. Her physical examination was normal, with no respiratory abnormalities.

The patient underwent a thoracoscopic right lobectomy. Intubation was performed using a 35 Fr left‐sided DLT. The anesthesiologist encountered resistance as the tracheal tube passed through the glottis. The operative time was 147 min, and the anesthesia time was 216 min. On postoperative day (POD) 1, the patient exhibited dyspnea and stridor. Percutaneous oxygen saturation was 93% with a nasal cannula set at an oxygen flow rate of 5 L/min. As the patient's respiratory status worsened in the general hospital room, the thoracic surgeons requested a Rapid Response System (RRS). The RRS anesthesiologist attempted to intubate the patient using a 7.0‐mm‐diameter endotracheal intubation tube, but subglottic resistance prevented successful intubation. The anesthesiologist was barely able to intubate the patient with a 6.5‐mm‐diameter endotracheal intubation tube. A tracheostomy was performed by the thoracic surgeons on POD 2. The patient was referred to our department for the evaluation and treatment of upper airway stenosis on POD 4.

## 3 Investigations and Treatment

**FIGURE 1:**
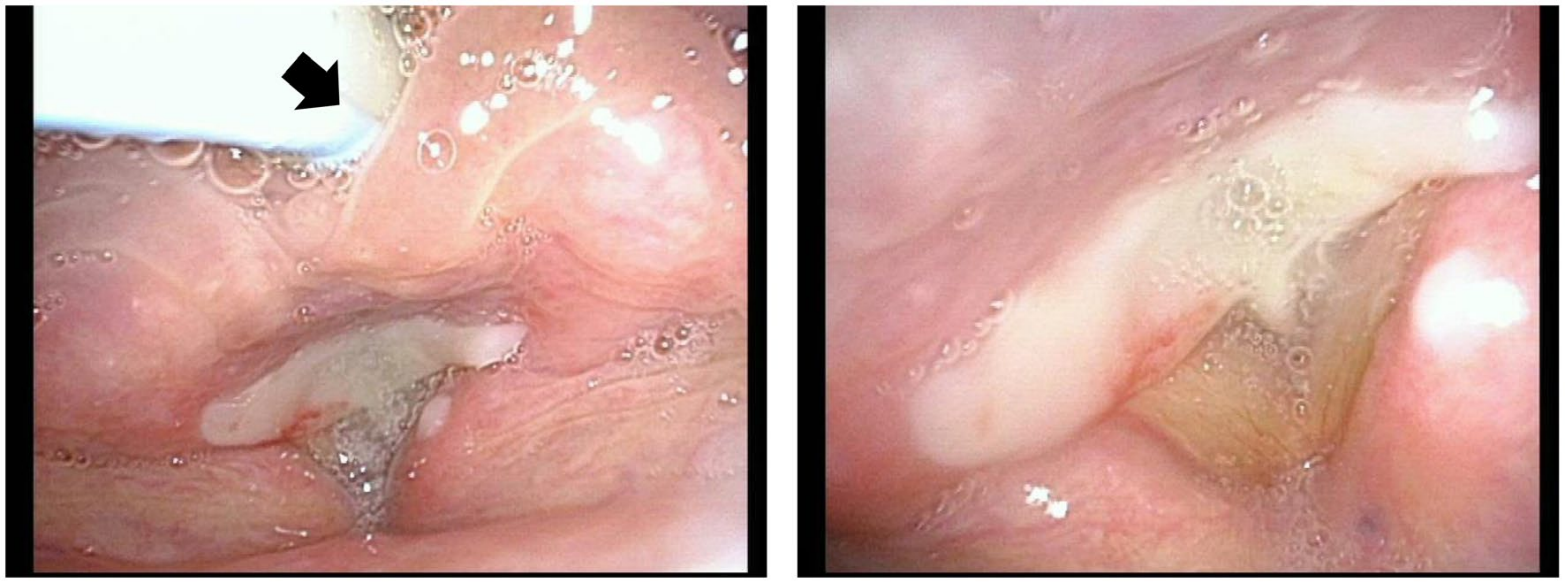
Local findings of POD 4. Laryngoscopy reveals complete obstruction of the glottis and posterior commissure with a thick white coat. Arrow indicates caudal. POD, postoperative day.

Laryngoscopy revealed a complete obstruction of the glottis and posterior commissure with a thick white coat (Figure 1). Based on the laryngeal findings, clinical course, and our experience with a similar case [7], we diagnosed subglottic stenosis after DTL intubation and traumatic laryngitis associated with reintubation. In consultation with the thoracic surgeons, we decided not to administer steroids and to observe the patient's condition. This was because the right lower lobectomy has a high risk of bronchial stump fistula [13], and steroid administration is associated with a risk of delayed wound healing.

## 4 Outcome and Follow‐Up

**FIGURE 2:**
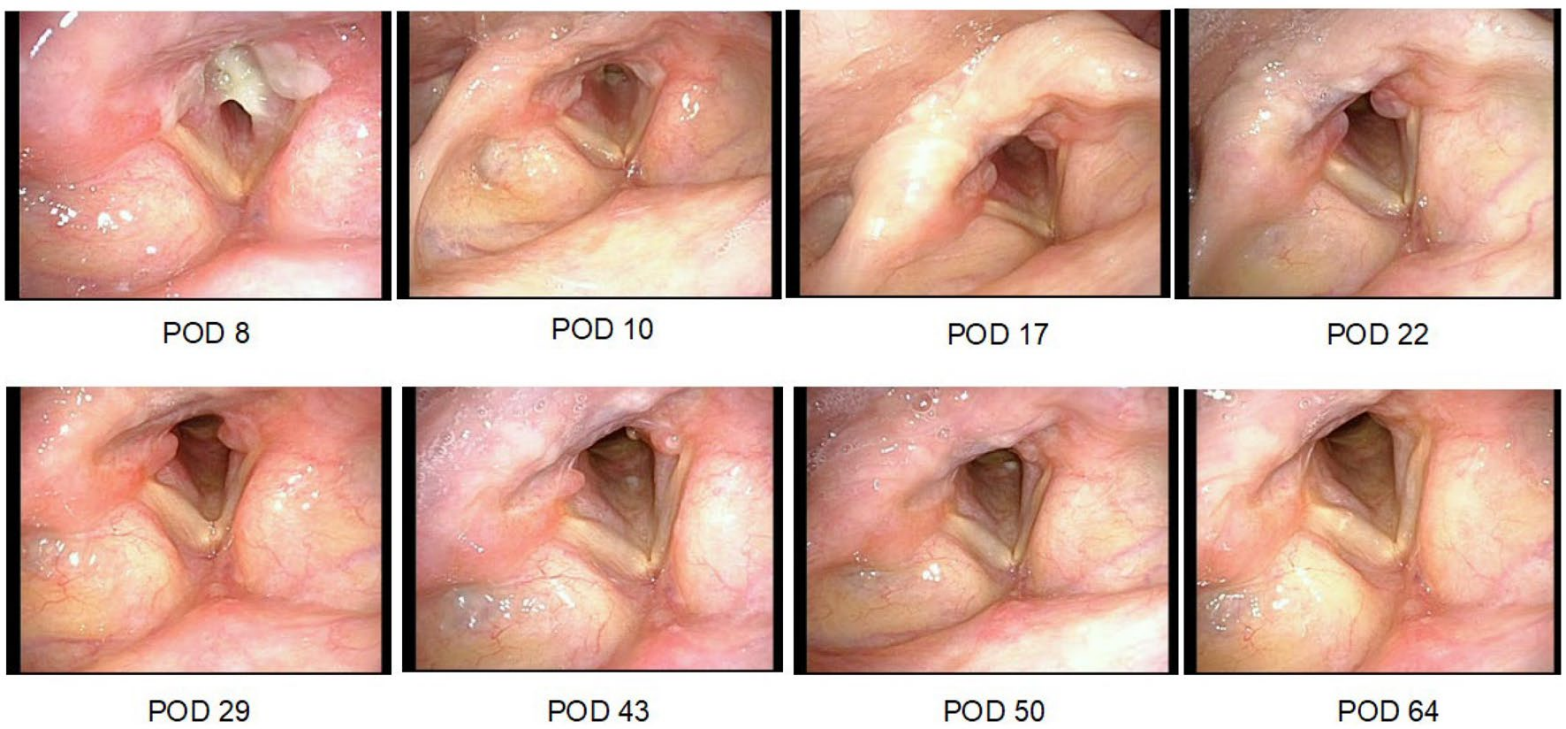
Later local findings from POD 8 to POD 64. POD, postoperative day.

The subglottic stenosis improved, and the patient was extubated on POD 17. By POD 22, the subglottic stenosis had resolved. However, bilateral masses over the vocal cord nodules, diagnosed as increasing laryngeal granulomas, appeared on the same day. The patient was treated with inhaled steroids (fluticasone propionate 200 μg/day). After 1 week of treatment, on POD 29, the granuloma showed hyperemia and the subglottic stenosis slightly worsened. The granuloma began to shrink, but a new mass appeared in the left subglottic region on POD 43. The left subglottic mass disappeared on POD 50, and the granuloma disappeared on POD 64 (Figure 2).

The patient is alive 7 months after surgery without subglottic stenosis and has no voice disorders.

## 5 Discussion

The cricoid cartilage is the narrowest part of the respiratory tract from the larynx to the trachea. It is the only complete cartilage ring in the airway, so it is not as flexible as the vocal cords and trachea [14]. A DLT is constructed by joining a bronchial tube and a tracheal tube, with the tracheal segment having a larger outer diameter than the bronchial segment. Therefore, after the bronchial segment passes through the cricoid cartilage, resistance may occur when the tracheal segment passes through the same area [7]. Repeated or forced intubation in the presence of resistance may cause subglottic damage.

The following three clinical issues are discussed: (1) the selection of an appropriate DLT diameter in elderly, short women, (2) the effect of steroids on subglottic stenosis after DLT intubation, and (3) reintubation of subglottic stenosis after DLT use.

Twelve cases of subglottic stenosis after DLT intubation at surgery, including the present case, have been reported. All patients were women over the age of 70 years, with nine of the 12 cases involving individuals shorter than 150 cm. A 35 Fr DLT was used in 11 cases, and a 32 Fr was used for initial intubation in one case involving a patient with a height of 140 cm (Table 1). Although small‐diameter DLTs reduce the risk of airway trauma, they complicate the suction and endoscopic manipulation and increase airway resistance. Therefore, it is desirable to use the largest possible DLT size; it is impossible to easily select a small DLT [7]. There is no consensus regarding the most practical, accurate, and useful method for predicting the size and depth of DLT insertion. Factors such as sex, height, tracheal diameter, cricoid cartilage diameter, and a one‐size fit have been reported to guide DLT selection in clinical practice [15]. Among these factors, sex and height are the most commonly used to guide the selection. For women shorter than 150 cm, the recommendation is to use 32 Fr DLT (Table 2) [16]. In Japan, a 35 Fr DLT is commonly used for women [17]. Sato and Kayashima [18] reported that a 35 Fr DLT was more likely to be oversized for Japanese women shorter than 150 cm. Oversized intubation is thought to be a cause of subglottic stenosis. Zolotenkova et al. [19] reported that in men, the area of cartilage tissue decreased with age, the cricoid cartilage was deformed, and the average number of chondrocytes and chondroblasts tended to decrease with age. The onset of the disease in people over the age of 70 years may be related to age‐related changes in the cricoid cartilage. Older, shorter women may need to be intubated with a DLT smaller than 35 Fr to avoid subglottic stenosis.

**TABLE 2:** Selection of tube size based on sex and height.

Male	> 170 cm	160–170 cm	< 160 cm
	41 Fr	39 Fr	37 Fr
Female	> 160 cm	150–160 cm	< 150 cm
	37 Fr	35Fr	32 Fr

Abbreviation: Fr, French.

Respiratory symptoms such as dyspnea and stridor due to subglottic stenosis occurred on POD 1 in this case and within POD 3 in all previously reported cases. Steroids were administered after the onset of respiratory symptoms in eight of the 11 previously reported cases. However, in all cases, the symptoms worsened, necessitating airway management. Subglottic stenosis is thought to be caused by mechanical irritation and subsequent inflammation [20]. Although steroids reduce inflammation, they are less effective at preventing the progression of subglottic stenosis after DLT intubation.

Reintubation of subglottic stenosis after DLT intubation is challenging and can induce new traumatic laryngitis. Reintubation was attempted in three of the 12 cases (Table 1), and in one case, the patient was unable to be intubated, resulting in cardiopulmonary arrest [4]. In two cases, including the present case, a 7.0‐mm‐diameter endotracheal intubation tube could not be used, and a smaller 6.5‐mm‐diameter endotracheal intubation tube was required due to intubation difficulty [5]. This case showed severe inflammation in the larynx compared to a previously reported case [7]. Subglottic stenosis may require a longer time to improve if reintubation is performed. Additionally, laryngeal granulomas developed, which may have been caused by reintubation. Considering that subglottic stenosis worsens after onset and postoperative management, such as sputum removal and ventilation, tracheostomy should be the first choice for airway management in cases of subglottic stenosis after DLT intubation.

In Asia, a 35 Fr DLT is commonly used for women, including women shorter than 150 cm [14, 21]. However, there have been no reports of subglottic stenosis after DLT intubation from other countries including Asia. It is unclear why this occurs only in Japan.

As the cricoid cartilage is the narrowest part of the respiratory tract from the larynx to the trachea, several literature reports suggest that measurement of the transverse diameter of the cricoid cartilage (TD‐C) by CT or ultrasound may be helpful in selecting the appropriate size of DLT [14, 18, 22]. Therefore, selection of the appropriate size of DLT based on the measurement of TD‐C may reduce the risk of subglottic stenosis after DLT intubation.

Shiqing et al. reported that the resistance encountered during DLT intubation was 52.5% when DLT size was selected based on CT measurements of TD‐C and left main bronchial equivalent diameter, and 72.5% when DLT size was selected based on CT measurements of left main bronchial equivalent diameter only in Asian women [23]. Resistance to intubation with the DLT is common, but if resistance is too severe to intubate, the DLT should be reduced to the next smaller size.

Subglottic stenosis after DTL intubation is likely to occur if the following conditions are met: resistance during a 35 Fr DLT intubation, repeated or forced intubation, older, shorter women.

The histopathology of the stenosis was not confirmed. Some limitations of this study include its small sample size and the predominant Japanese patient population in the available literature. Further research is warranted to optimize management strategies for DLT intubation.

## 6 Conclusion

Subglottic stenosis after DLT intubation is a significant challenge. This report highlights the need for cautious airway management when intubating a 35 Fr DLT, particularly in older, shorter women. Respiratory symptoms appear within POD 3. Steroids are thought to be less effective in suppressing the progression of subglottic stenosis. Tracheostomy emerges as a primary consideration in the management of subglottic stenosis after DLT intubation. Further research is warranted to optimize management strategies for DLT intubation.

## Author Contributions

**Masami Suzuki:** project administration, writing – original draft. **Naohiro Yoshida:** project administration, writing – review and editing.

## Consent

Written informed consent was obtained from the patient to publish this report in accordance with the journal's patient consent policy.

## Conflicts of Interest

The authors declare no conflicts of interest.

## Data Availability

The data that support the findings of this study are available from the corresponding author upon reasonable request.
